# Operator Radiation Exposure During Transfemoral Transcatheter Aortic Valve Replacement

**DOI:** 10.1016/j.shj.2022.100002

**Published:** 2022-03-25

**Authors:** Sunny Goel, Richard Casazza, Ravi Teja Pasam, Enrico Montagna, Joseph Gotesman, Robert Frankel, Elliot Borgen, Gregory Crooke, Paul Saunders, Jacob Shani

**Affiliations:** a Department of Cardiology, Mount Sinai Medical Center, New York, New York, USA; b Department of Cardiology, Maimonides Medical Center, Brooklyn, New York, USA; c Department of Internal Medicine, Lahey Hospital and Medical Center, Burlington, Massachusetts, USA; d Department of Cardiothoracic Surgery, Maimonides Medical Center, Brooklyn, New York, USA

**Keywords:** Obesity, Radiation exposure, TAVR

## Abstract

**Background:**

The level of radiation exposure received by operators performing transcatheter aortic valve replacement (TAVR) is not well investigated. The aim of this study is to measure the amount of radiation received by operators performing transfemoral TAVR and to identify various patient and procedural characteristics associated with increased radiation exposure.

**Methods:**

Primary (operator 1) and secondary (operator 2) operators' equivalent radiation doses in micro Sieverts (µSv) were calculated prospectively using real-time radiation dosimeters for a total of 140 consecutive transfemoral TAVRs. Corresponding eye and thorax radiation exposures between the operators were compared. Associations between various patient and procedural characteristics and the radiation exposure were tested using the t-test and Wilcoxon Mann-Whitney rank-sum test with Monte Carlo estimation. Multivariable regression analysis was also conducted.

**Results:**

Operator 1 had significantly higher cumulative equivalent radiation exposure than operator 2 (86 µSv vs 38 µSv, *p*-value: <0.0001) which was consistent at the level of the thorax (67 µSv vs 22 µSv, *p*-value: <0.0001), but not at the level of the eye (16.5 µSv vs 15 µSv, *p*-value: 0.30). On multivariable analysis, patient obesity and intraprocedural complications were associated with higher radiation exposure to both operators. Ad hoc percutaneous coronary intervention led to excessive radiation exposure to the secondary operator.

**Conclusions:**

Transfemoral TAVR is associated with a modest amount of radiation exposure to operators and is significantly higher for the primary operator than for the secondary operator.

## Introduction

Fluoroscopy is an indispensable tool for interventional cardiologists to treat patients with coronary artery disease and valvular abnormalities. However, exposure to ionizing radiation during fluoroscopic procedures poses a significant health hazard to physicians and ancillary staff. The multitude of effects of radiation exposure from fluoroscopy to interventional cardiologists is well known.^1^ Radiation exposure may lead to cataracts, skin and hair changes, accelerated vascular aging, and early atherosclerosis.^2^^,^^3^ There have also been case reports of left-sided brain tumors in interventional cardiologists, raising concerns for an association between radiation exposure and brain malignancy.^4^

The last decade has seen exponential growth in the space of percutaneous valvular therapeutics, with transcatheter aortic valve replacement (TAVR) leading among such procedures. During TAVR, fluoroscopic guidance is used for valve assessment, valve deployment, and access site management. Previous studies have reported that the amount of radiation exposure to the patient undergoing TAVR is equivalent to the radiation received during a moderate complexity percutaneous coronary intervention (PCI).^5^ However, the amount of radiation received by the operators performing TAVR is not well studied. With expansion of TAVR to younger and low-risk surgical patients, it is important to quantify the amount of radiation received by the operators performing this complex procedure. In addition, it is critical to investigate the patient- and procedure-specific risk factors that are associated with increased radiation dose exposure to the operators. Therefore, we undertook this study to evaluate the amount of radiation received by operators performing transfemoral TAVR.

## Methods

### Study Design

This is a prospective, observational, single-center study conducted at Maimonides Medical Center, Brooklyn, USA. The study protocol was approved by the institutional review board. Informed consent was obtained from all patients. All consecutive transfemoral TAVR procedures from September 2017 to October 2019 were included in the study. The study was funded by the spring 2017 Maimonides Research and Development Grant.

### Patient Selection and Study Operators

The patient candidacy, access site, and valve type were decided by the structural heart team based on imaging studies and patient clinical factors. Interventional cardiologists, cardiac surgeons, and imaging specialists were part of the structural heart team. The operator performing the majority of the procedure, standing in the first position, was considered as the primary operator (operator 1), and the operator who assisted the primary operator, standing in the second position, was considered as the secondary operator (operator 2). A total of 3 interventional cardiologists and 2 cardiothoracic surgeons, all proficient with transfemoral TAVR, performed all the study procedures.

### Study Procedure

All procedures were performed in a hybrid operating room, under fluoroscopic and echocardiography imaging guidance, in a standard fashion. All operators wore standard radiation protection equipment, consisting of a leaded vest, skirt, lead glasses, and a thyroid shield. A lead ceiling-mounted shield with a patient contour cutout and a table-mounted lead shield attached to the side of the operating table extending from the table to the floor were present. No RADPAD or Sentinel protection devices were used. The hybrid operating room was equipped with a Siemens Artis zeego system (Siemens, Munich, Germany). ALARA (as low as reasonably achievable) radiation practices were employed for the study. All procedures were conducted using 7.5 frames per second fluoroscopy setting, with minimal cineangiography, using optimal collimation while maintaining a minimal distance between the patient and the image detector, using a maximal source to image distance, and limiting magnification. The C-arm unit was kept in the coplanar angles for valve deployment, and operators utilized posterior-anterior projection and/or slightly right anterior oblique projections for the majority of the procedure to decrease the amount of radiation exposure.

### Radiation Measurement

**Figure 1:**
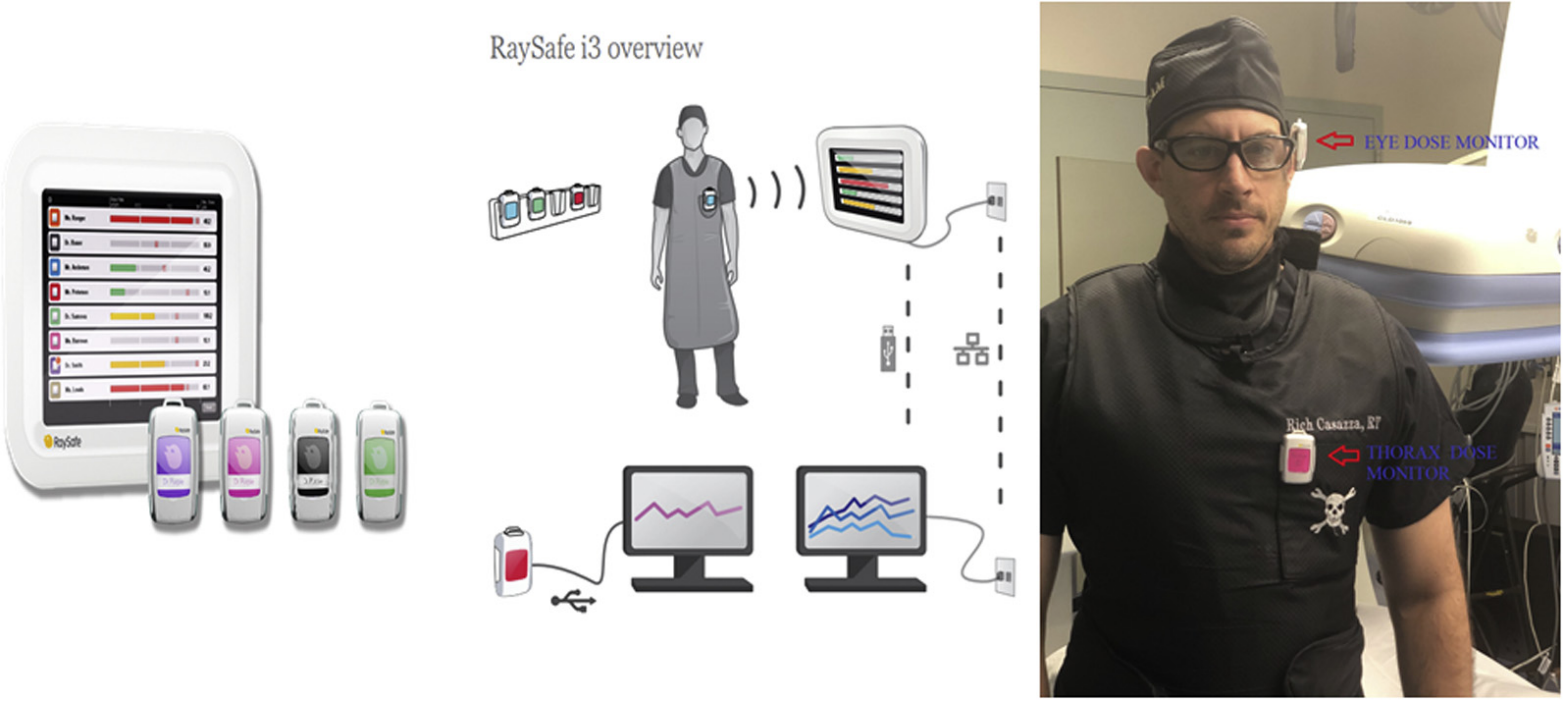
**Radiation measurement using real-time dosimeters**.

Operator radiation exposure was assessed with a set of 2 electronic Raysafe i3 Real-time Radiation Dosimeters (Billdal, Sweden), one strategically placed on the thorax over the lead apron pocket and the other one on the left anterior side of the protection eye glass. The radiation dose was measured in microsieverts (μSv). The dosimeter data were sent to a bedside monitor capable of displaying real-time radiation doses [Figure 1]. Operators were blinded to the monitor display and to the radiation data collected by the dosimeters for the duration of the study.

Patient radiation metrics including the fluoroscopy time (FT) and dose area product (DAP) were automatically calculated by the fluoroscopic imaging system. Postprocedurally, DAP-normalized and FT-normalized equivalent doses for operators were calculated to account for these factors.

Total cumulative radiation doses, i.e., the combined thorax and eye radiation dose received by the operators, as well as cumulative radiation doses to the thorax and eye per procedure for individual operators, were measured. After correction for the radiation weighting factor (for x-rays, this factor is 1), the results were expressed as equivalent doses in μSv. The equivalent dose at the thorax was also converted to an operator effective dose by dividing it by a factor of 33 as per apron thickness (0.5-mm lead equivalent and with tube voltage under the table).^6^ The DAP-normalized equivalent dose and FT-normalized equivalent dose were calculated by dividing the equivalent dose by the DAP and FT, respectively. We also analyzed the patient and procedural variables, including use of transesophageal echocardiogram, predilation, postdilation, patient obesity (body mass index [BMI] >30 kg/m^2^), left main protection, intraprocedural complications requiring fluoroscopy guidance, TAVR valve types, and horizontal aorta (annular angle >60 degree based on computed tomography scan) which could possibly affect the amount of radiation received by the operators.

### Statistical Analysis

Descriptive statistics including frequencies, percentages, mean, median, standard deviation, and first and third quartile numbers were reported for baseline characteristics and radiation dose measurements. The Shapiro-Wilk test was used to assess for normal distribution of radiation doses and exposure variables. A *p*-value of less than 0.05 indicates that the data are not normally distributed. If the data were normally distributed, the t-test was used, and in non-normal distribution, the exact Wilcoxon Mann-Whitney rank-sum test with Monte Carlo estimation was utilized. The one-sided *p*-value was used for all variables except for the TAVR valve type.

Furthermore, patient and procedural variables that had a *p*-value of 0.20 or less were included for multivariable regression analysis. Log-transformation of radiation was performed for the purpose of regression analysis as it did not follow a normal distribution. The log-transformed data were again checked for normal distribution with the abovementioned statistical tests. The coefficients from this analysis were then converted to interpret table parameter estimates by exponentiating the coefficients. A *p*-value of less than 0.05 in the multivariable regression analysis was considered significant association with the operator radiation exposure.

## Results

A total of 140 patients underwent transfemoral TAVR during the study period. Table 1 describes the baseline and procedural characteristics. The median age of patients was 81.5 years, and 57.1% of subjects were female. Edwards’ balloon-expandable Sapien 3 valve was used in 52.9% patients, and Medtronic’s self-expandable Evolut R/Evolut Pro valve was used in 47.1% patients. The mean BMI of patients undergoing TAVR was 27.9 kg/m^2^. The median FT was 18.9 minutes. Two patients required left main protection with one of them needing chimney stenting of left main coronary artery. Sixteen patients (11.4%) had intraprocedural complications with the access site vascular complication being the commonest. Four patients required percutaneous transluminal angioplasty, 5 patients needed covered stent placement, and 3 patients necessitated open surgical repair. The nonvascular complication included one patient with ventricular fibrillation after valve deployment requiring the patient to go on cardiopulmonary bypass emergently and subsequent stenting of the left main artery. One patient had a high CoreValve implant (final position above the aortic annulus) requiring another CoreValve to stabilize the prosthesis successfully. Two patients required an emergent pericardial window owing to perforation.

**Table 1:** Baseline and procedural characteristics

Total number	140
Female sex, n (%)	80 (57.1)
Age in y, median (Q1, Q3)	81.5 (76.5, 88.0)
BMI in kg/m^2^, median (Q1, Q3)	27.9 (25.2, 32.5)
BSA in m^2^, median (Q1, Q3)	1.8 (1.6, 1.9)
DAP in cGy × cm^2^, median (Q1, Q3)	7867.5 (5122.2, 13,891.5)
Total contrast used in ml, mean ± SD	114.61 ± 51.71
Total fluoroscopy time in min, median (Q1, Q3)	18.9 (14.75, 25.80)
Balloon-expandable valve, n (%)	74 (52.9)
20 mm	2 (2.7)
23 mm	37 (50.0)
26 mm	27 (36.5)
29 mm	8 (10.8)
Self-expandable valve, n (%)	66 (47.1)
23 mm	5 (7.5)
26 mm	29 (43.9)
29 mm	28 (42.4)
34 mm	4 (6.1)
Valve area in cm^2^, mean +SD	0.77 + 0.22
Time to cross the valve in min, median (Q1, Q3)	1.5 (0.5, 4)
Use of TEE, n (%)	25 (17.8)
Left main protection needed, n (%)	2 (1.4)
Intraprocedural complications, n (%)	16 (11.4)
Predilation, n (%)	76 (54.2)
Postdilation, n (%)	38 (27.1)
Valve in valve TAVR, n (%)	3 (2.1)
Bicuspid aortic stenosis, n (%)	2 (1.4)
Horizontal aorta, n (%)	13 (9.2)

BMI = body mass index, BSA = body surface area, DAP = dose area product, TAVR = transcatheter aortic valve replacement, TEE = transesophageal echocardiogram.

**Figure 2:**
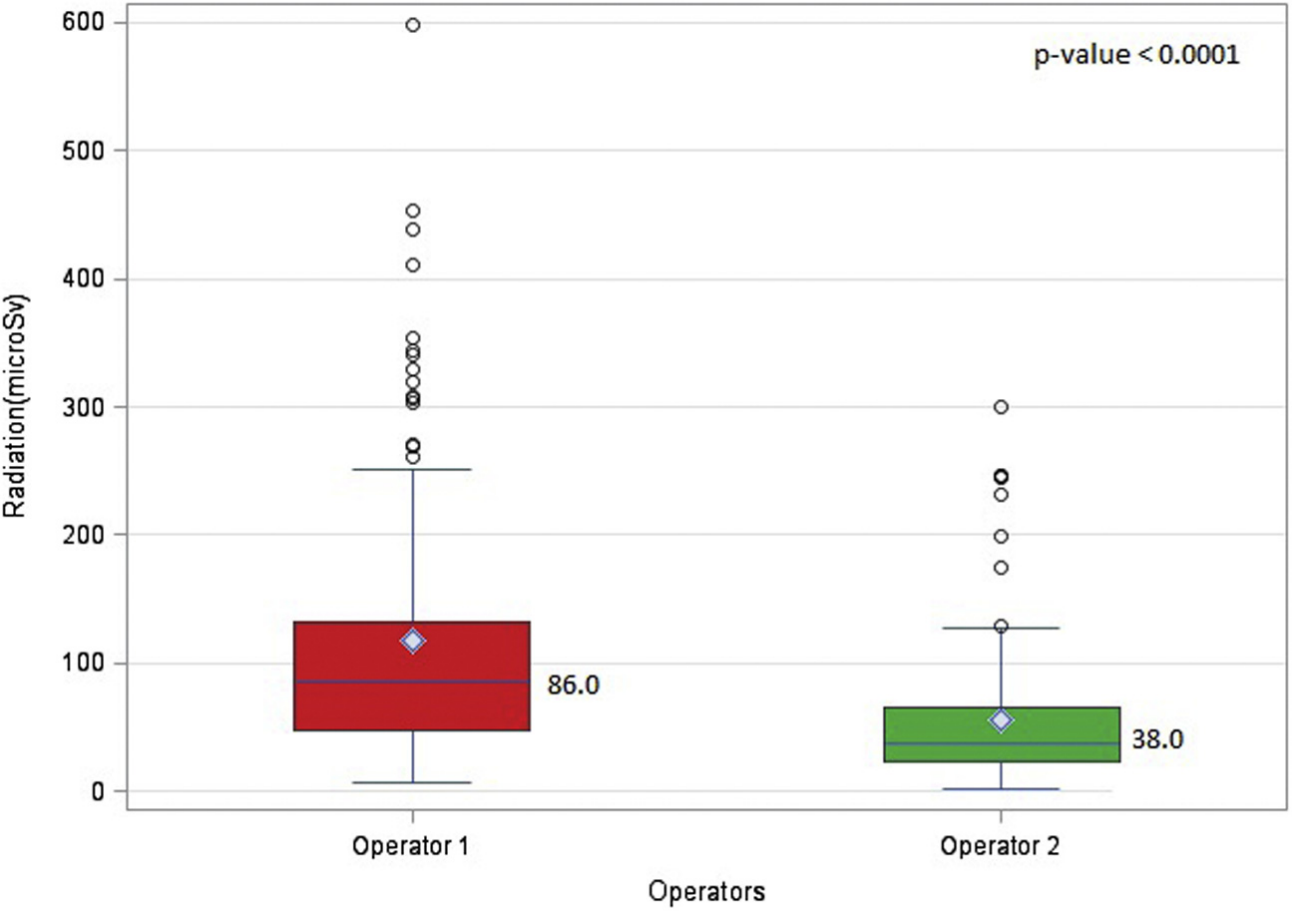
**Total equivalent radiation dose for operator 1 vs. operator 2 performing transfemoral TAVR.** (The diamond denotes the mean. The top and the bottom ends denote the first and third quartiles. The whiskers are the maximum and minimum values closest to the upper and lower fence. The upper fence is obtained by adding the value of 1.5 times the interquartile range added to the third quartile. The lower fence is obtained by subtracting 1.5 times the interquartile range from the first quartile. The circles denote the outliers that are beyond the fences). Abbreviation: TAVR, transcatheter aortic valve replacement.

**Figure 3:**
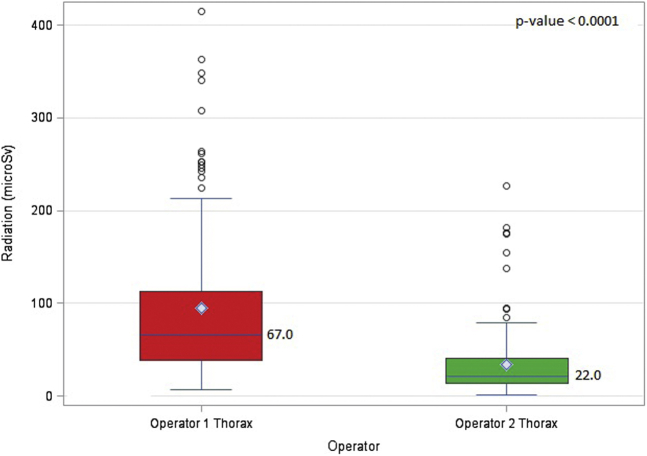
**Total equivalent radiation dose for operator 1 vs. operator 2 at the thorax level.** (The diamond denotes the mean. The top and the bottom ends denote the first and third quartiles. The whiskers are the maximum and minimum values closest to the upper and lower fence. The upper fence is obtained by adding the value of 1.5 times the interquartile range added to the third quartile. The lower fence is obtained by subtracting 1.5 times the interquartile range from the first quartile. The circles denote the outliers that are beyond the fences).

**Figure 4:**
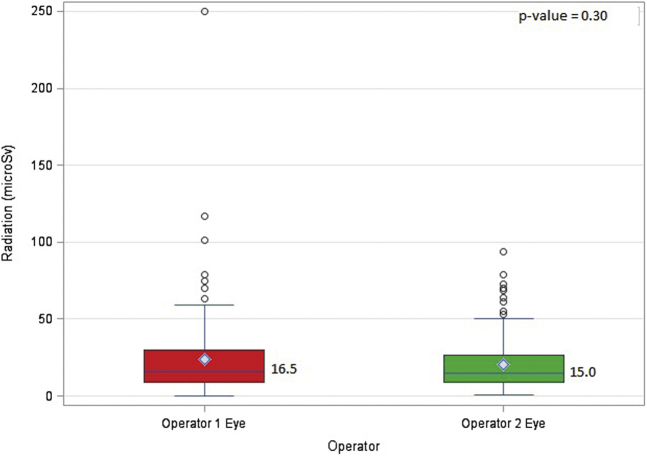
**Total equivalent radiation dose for operator 1 vs. operator 2 at the eye level.** (The diamond denotes the mean. The top and the bottom ends denote the first and third quartiles. The whiskers are the maximum and minimum values closest to the upper and lower fence. The upper fence is obtained by adding the value of 1.5 times the interquartile range added to the third quartile. The lower fence is obtained by subtracting 1.5 times the interquartile range from the first quartile. The circles denote the outliers that are beyond the fences).

Operator 1 had a significantly higher cumulative equivalent radiation dose than operator 2 (86 μSv vs. 38 μSv, *p*-value: <0.0001) [Figure 2, Table 2]. Also, operator 1 had a higher equivalent radiation dose to the thorax than operator 2 (67 μSv vs. 22 μSv, *p*-value: <0.0001) [Figure 3] (Table 3). The effective dose received by operator 1 (2.03 μSv vs. 0.66 μSv, *p*-value: <0.0001), DAP-normalized equivalent doses (0.011 μSv/cGYcm^2^ vs. 0.005 μSv/cGYcm^2^, *p*-value: <0.0001), and FT-normalized equivalent doses (4.35 μSv/min vs. 2.05 μSv/min, *p*-value: <0.0001) were also significantly higher than those received by operator 2 (Table 3). Nevertheless, no parameters were significantly different between the operators for eye exposure [Figure 4] (Table 4).

**Table 2:** Total equivalent radiation dose for operator 1 vs. operator 2 including the DAP and FT normalized

Operator	Radiation (μSv), median (first quartile-third quartile)	*p*-value	DAP-normalized radiation (μSv/cGYcm^2^), median (first quartile-third quartile)	*p*-value	FT-normalized radiation (μSv/min), median (first quartile-third quartile)	*p*-value
Operator 1	86 (48-133)	<0.0001	0.011 (0.007-0.015)	<0.0001	4.35 (2.65-6.99)	<0.0001
Operator 2	38 (23-65)		0.005 (0.003-0.007)		2.05 (1.30-3.24)	

DAP = dose area product, FT = fluoroscopy time.

**Table 3:** Total equivalent radiation dose for operator 1 vs. operator 2 at the thorax level including the DAP and FT normalized

Operator	Radiation (μSv), median (first quartile-third quartile)	*p*-value	DAP-normalized radiation (μSv/cGYcm^2^), median (first quartile-third quartile)	*p*-value	FT-normalized radiation (μSv/min), median (first quartile-third quartile)	*p*-value
Operator 1 thorax	67 (39-112.5)	<0.0001	0.009 (0.006-0.013)	<0.0001	3.42 (2.09-5.44)	<0.0001
Operator 2 thorax	22 (14-41)		0.003 (0.002-0.004)		1.18 (0.79-1.90)	

DAP = dose area product, FT = fluoroscopy time.

**Table 4:** Total equivalent radiation dose for operator 1 vs. operator 2 at the eye level including the DAP and FT normalized

Operator	Radiation (μSv), median (first quartile-third quartile)	*p*-value	DAP-normalized radiation (μSv/cGYcm^2^), median (first quartile-third quartile)	*p*-value	FT-normalized radiation (μSv/min), median (first quartile-third quartile)	*p*-value
Operator 1 eye	16.5 (9-30)	0.30	0.0019 (0.0012-0.0028)	0.25	0.88 (0.46-1.33)	0.35
Operator 2 eye	15 (9-26.5)		0.0018 (0.0013-0.0027)		0.76 (0.48-1.29)	

DAP = dose area product, FT = fluoroscopy time.

On multivariable regression analysis, patient obesity and intraprocedural complication were associated with a higher radiation exposure to both operators (Table 5). Obese patients and patients who have had complications led to an increase in radiation exposure to operator 1 by 1.69 μSv and 1.58 μSv, respectively (Table 5). Obese patients, patients who have had complications, and those who required ad hoc PCI led to an increase in radiation exposure to operator 2 by 1.56 μSv, 2.15 μSv, and 3.52 μSv, respectively (Table 5). The results of univariate analysis for various patient and procedural variables are described in the supplementary material (Supplemental Tables 1-3). The multivariable regression analysis including the FT is also described in the supplementary material (Supplemental Table 4).

**Table 5:** Multivariable regression analysis of effect of various patient and procedural factors on radiation exposure to operators 1 and 2

Variable	Parameter estimate∗	95% confidence intervals†	*p*-value
Operator 1			
Obesity	1.69	1.30-2.20	0.0001
Complications	1.58	1.07-2.34	0.02
Operator 2			
Obesity	1.56	1.19-2.04	0.001
Ad hoc PCI	3.52	1.21-10.23	0.02
Complications	2.15	1.44-3.22	0.0003

*Notes*. Multivariable regression analysis using log-transformed values of radiation for operator 1.

PCI = percutaneous coronary intervention.

∗ Parameter estimates derived from the coefficients from the log-transformed data by exponentiating the coefficients.

† 95% confidence intervals were also obtained from the abovementioned formula after adding and subtracting the value of 1.96 times the standard error to the log-transformed data.

## Discussion

The key findings of our study are summarized as follows: 1. The effective radiation dose received by operators performing transfemoral TAVR was modest; 2. Overall, operator 1 had a significantly higher equivalent radiation dose than operator 2; 3. Compared to operator 2, operator 1 had a significantly higher radiation exposure to the thorax, but this difference was not noted for the radiation exposure to the eye; 4. Patient obesity and intraprocedural complications were associated with a higher radiation exposure to operators.

The development and refinement of advanced invasive cardiovascular procedures over the past 2 decades have led to increased radiation exposure to both patients and medical personnel. Radiation exposure to operators performing TAVR is ill-defined, and now, with expansion of TAVR to lower-risk and younger patients, the volume of procedures performed by operators is bound to increase. With this study, we were able to demonstrate that the operators performing transfemoral TAVR were exposed to modest amounts of effective radiation (2.03 μSv for operator 1 and 0.66 μSv for operator 2) which is on par with operators performing diagnostic angiography and/or PCI (an average effective dose of 1.2 μSv per procedure for femoral access and 2.3 μSv for radial access).^6^ A busy interventional cardiologist practicing the ALARA principle and donning proper radiation protective equipment receives approximately 2-4 mSv/y, with dose depending upon time in the laboratory and case complexity.^7^, ^8^, ^9^ Transfemoral TAVR, a complex interventional procedure, requires fluoroscopy guidance for several steps, such as vascular access, sheath placement, obtaining coplanar angles, TAVR valve assessment, sentinel device placement, valve crossing, balloon valvuloplasty, valve positioning, deployment, and vascular closure, and is expected to expose operators to high amounts of radiation exposure. However, in our study, we did not find operators to be exposed to excessive radiation. This may be explained by the fact that there is superior preprocedural planning with TAVR in terms of navigating access site challenges (femoral artery calcification, peripheral vascular tortuosity) and upfront knowledge about anatomical challenges (prosthetic valve, bicuspid valve, porcelain aorta, horizontal aorta, heavily calcified valve, left ventricular outflow tract calcium). In addition, there is less use of cine fluoroscopy and steep angular projections during TAVR procedures, which are common in PCI procedures. Moreover, there is constant echocardiography support during TAVR, which reduces the sole reliance on fluoroscopy and, therefore, reduces radiation exposure to operators. The National Council on Radiation Protection and Measurements recommends an effective dose limit of 50 mSv per year, and a cumulative lifetime dose of 10 mSv multiplied by the age for radiation workers, and 1 mSv per year for the general public.^10^ Considering that a busy operator is performing an average of 100 TAVRs per year, the total effective radiation dose per year will be <1 mSV, which is considerably lower than the effective dose limit. However, operators must be mindful of the total dose accumulated over years and the stochastic effects of radiation, which are dose independent.

Radiation exposure is inversely proportional to the square of the distance from the radiation source.^11^ Operator 1, being in close proximity to the radiation source, understandably receives a greater amount of cumulative total and thorax radiation exposure than operator 2. However, no significant difference in eye exposure was found between the 2 operators. This may be explained by the fact that the distance traveled by the radiation beam to the operator’s eye is greater and therefore subject to far more scattering. This leads to overall reduced radiation exposure to operators at the eye level. In addition, operator 2 frequently leans their head to the table center (radiation source) to observe the procedure and, unlike the primary operator, has no ceiling-mounted protecting lead glass. Hence, we theorize, operator 2 is exposed to a higher radiation exposure at the eye level. However, this hypothesis must be studied in a prospective manner to confirm its accuracy.

The greatest source of physician radiation exposure during cardiac catheterization comes from scatter radiation emitted from the patient.^12^ The patient BMI has been clearly shown to impact the patient radiation dose during fluoroscopic procedures.^13^ Because greater radiation doses are required to produce adequate images in obese patients and there is difficulty in optimal positioning of accessory lead shields in morbidly obese patients, greater amounts of scatter radiation are emitted. We, too, found that obesity among patients is associated with a higher radiation exposure to operators even after normalizing the operator radiation dose to the DAP and FT.

Use of left main protection in TAVR requires additional steps such as guide engagement, coronary wiring, and/or placing an undeployed stent in the left main artery which all require fluoroscopy guidance and, hence, contribute to greater amounts of radiation exposure to operators as demonstrated by our study. However, on multivariate analysis, this was significant only for the secondary operator. Similarly, intraprocedural complications, especially vascular, almost always require fluoroscopy guidance for rectification (percutaneous transluminal angioplasty, covered stents), thus exposing operators to an excessive radiation dose as appreciated in our study.

The equivalent dose for operator 1 at the level of the eye was 16.5 μSv in our study, whereas it has been shown to be 30 μSv in a previous study.^14^ This difference might be attributed to new-generation valves with better equipment and an improved delivery system, fewer complications, and the operators becoming proficient in performing TAVR, thereby cutting down on accumulated radiation doses. However, lack of data on FT in the other study prevents us from making this conclusion affirmatively. On the other hand, the results from our study are in line with the recently published studies evaluating the operator radiation exposure during TAVR.^15^^,^^16^ However, our study sample is larger, is more uniform (only transfemoral TAVR), comparing the total equivalent radiation exposure to the operators including the eye radiation exposure, and also evaluates the various patient and procedural factors that can affect the operator radiation dose.

### Study Limitations

1. Although this is the largest published series of radiation exposure to operators during TAVR to date, the number of cases is relatively small and represents the experience of one academic center with only a few operators;
2. We did not use sentinel devices for cerebral protection which require additional fluoroscopy guidance and would have added to the total radiation doses;
3. Our cardiac catheterization laboratory does not use RADPAD radiation protection pads which have shown to reduce radiation doses to the operators, so we could not comment on whether or not this may have reduced the total radiation exposure to the operators;
4. No tube angulations during valve deployment were recorded for this study. Steep angulations have shown to increase radiation exposure to the operators. Hence, no conclusions may be made in regard to angulations;
5. No other TAVR approaches were studied for radiation exposure to operators. However, transfemoral approach is currently the most common and routinely used access used for TAVR;
6. In all cases, the main access site closure was completed using dry closure with balloon occlusion of puncture site after the deployment of the vascular closure device Perclose (Abbott Vascular Devices, CA), which required fluoroscopy assistance to correctly position the balloon. We cannot remark on whether or not dry closure technique added significant radiation exposure to the operators.

## Conclusion

Our study demonstrated that the effective radiation dose received by operators during TAVR is reasonably low and should be reassuring to operators performing this complex procedure. In total and at the level of the thorax, operator 1 is exposed to higher amounts of radiation than operator 2, but this difference is not observed at the level of the eye. Patient obesity and intraprocedural complications were associated with a higher radiation exposure to operators.

## Ethics Statement

This research article has adhered to the relevant ethical guidelines. The study protocol was approved by the institutional review board of Maimonides Medical Center. Informed consent was obtained from all patients.

## Funding

The authors have no funding to report.

## Disclosure statement

The authors report no conflict of interest.
