# Supplementary material for: Operator Radiation Exposure During Transfemoral Transcatheter Aortic Valve Replacement
Source: Struct Heart. 2022 Mar 25;6(1):100002. doi: 10.1016/j.shj.2022.100002 (PMC10236896; doi:10.1016/j.shj.2022.100002)
Supplement: Supplemental Tables 1-4 [file mmc1.docx]

**SUPPLEMENTARY MATERIAL**

**Supplementary Table 1- Effect of various patient and procedural factors on radiation exposure to operators**

| **Variable** | **Operator 1 (µSv)**, median (1^st^ quartile – 3^rd^ quartile) | **p-value** | **Operator 2 (µSv)**, median (1^st^ quartile – 3^rd^ quartile) | **p-value** |
| --- | --- | --- | --- | --- |
| **TEE** |  | | | |
| Yes | 110 (74-231) | 0.04 | 48 (32-102) | 0.02 |
| No | 81 (47-131) |  | 37 (37-96) |  |
| **Pre dilation** |  | | | |
| Yes | 85 (49-127.5) | 0.48 | 35 (19.5-64.5) | 0.08 |
| No | 92 (46-139) |  | 40 (29-71.5) |  |
| **Post dilation** |  | | | |
| Yes | 74 (50-133) | 0.30 | 37.5 (25-80) | 0.22 |
| No | 90 (47-133) |  | 38 (22-64) |  |
| **Obesity** |  | | | |
| Yes | 113 (74-233) | 0.0001 | 51 (32-96) | 0.002 |
| No | 75 (44-109) |  | 33 (20-60) |  |
| **Left main protection** |  | | | |
| Yes | 265 (119-411) | 0.049 | 72 (159-246) | 0.03 |
| No | 85 (47-133) |  | 37 (23-64) |  |
| **Intra- procedural Complications** |  | | | |
| Yes | 116.5 (88-234) | 0.03 | 61 (36.5-149 | 0.002 |
| No | 80 (47-125.5) |  | 35 (21.5-62.5) |  |
| **Valve Type** |  | | | |
| Edwards | 80 (42-125) | 0.31 (Two-sided) | 33 (18-64) | 0.08 (Two-Sided) |
| Medtronic | 95.5 (54-145) |  | 43 (29-66) |  |
| **Horizontal aorta** |  |  |  |  |
| Yes | 89 (73-109) | 0.06 | 52 (31-64) | 0.24 |
| No | 81 (46-133) |  | 37 (22-65) |  |

**Supplementary Table 2- DAP normalized radiation dose for patient and procedural variables**

| **Variable** | **Operator 1 (µSv/cGYcm²)**, median (1^st^ quartile – 3^rd^ quartile) | **p-value** | **Operator 2 (µSv/cGYcm²)**, median (1^st^ quartile – 3^rd^ quartile) | **p-value** |  |
| --- | --- | --- | --- | --- | --- |
| **TEE** |  | | | | |
| Yes | 0.009 (0.006-0.012) | 0.02 | 0.005 (0.003-0.006) | 0.15 |  |
| No | 0.011 (0.008-0.016) |  | 0.005 (0.003-0.007) |  |  |
| **Predilation** |  | | | | |
| Yes | 0.011 (0.008-0.015) | 0.21 | 0.004 (0.003-0.007) | 0.06 |  |
| No | 0.010 (0.007-0.015) |  | 0.006 (0.003-0.007) |  |  |
| **Post dilation** |  | | | | |
| Yes | 0.010 (0.007-0.013) | 0.12 | 0.005 (0.004-0.007) | 0.15 |  |
| No | 0.012 (0.007-0.016) |  | 0.005 (0.003-0.007) |  |  |
| **Obesity** |  | | | | |
| Yes | 0.011 (0.008-0.017) | 0.005 | 0.006 (0.004-0.008) | 0.0003 |  |
| No | 0.010 (0.006-0.012) |  | 0.004 (0.003-0.006) |  |  |
| **Left main protection** |  | | | | |
| Yes | 0.008 (0.005-0.011) | 0.19 | 0.005 (0.003-0.007) | 0.49 |  |
| No | 0.11 (0.007-0.15) |  | 0.005 (0.003-0.007) |  |  |
| **Complications** |  | | | | |
| Yes | 0.011 (0.007-0.015) | 0.47 | 0.004 (0.006-0.009) | 0.05 |  |
| No | 0.011 (0.007-0.016) |  | 0.005 (0.003-0.007) |  |  |
| **Valve Type** |  | | | | |
| Edwards | 0.011 (0.007-0.017) | 0.32 | 0.005 (0.003-0.007) | 0.86 |  |
| Medtronic | 0.010 (0.007-0.013) |  | 0.005 (0.003-0.006) |  |  |
| **Horizontal Aorta** |  |  |  |  |  |
| Yes | 0.011 (0.008-0.0014) | 0.24 | 0.005 (0.004-0.006) | 0.51 |  |
| No | 0.011 (0.007-0.0015) |  | 0.005 (0.003-0.007) |  |  |

**Supplementary Table 3-Fluoroscopy time normalized radiation dose for patient and procedural variables**

| **Variable** | **Operator 1 (µSv/min)** , median (1^st^ quartile – 3^rd^ quartile) | **p-value** | **Operator 2 (µSv/min)** , median (1^st^ quartile – 3^rd^ quartile) | **p-value** |
| --- | --- | --- | --- | --- |
| **TEE** |  | | | |
| Yes | 5.02 (2.97-7.66) | 0.20 | 2.79 (1.41-4.67) | 0.10 |
| No | 4.33 (2.61-6.61) |  | 2.01 (1.30-3.10) |  |
| **Pre dilation** |  | | | |
| Yes | 4.13 (2.65-7.06) | 0.44 | 1.78 (1.13-2.83) | 0.04 |
| No | 4.66 (2.79-6.91) |  | 2.26 (1.50-3.33) |  |
| **Post dilation** |  |  |  |  |
| Yes | 4.06 (2.66-5.39) | 0.11 | 1.84 (1.43-2.90) | 0.44 |
| No | 4.52 (2.64-7.44) |  | 2.016(1.28-3.30) |  |
| **Obesity** |  | | | |
| Yes | 6.53 (3.99-10.47) | <0.0001 | 2.97 (1.79-4.16) | <0.0001 |
| No | 3.64 (2.38-5.09) |  | 1.65 (1.18-2.50) |  |
| **Left main protection** |  | | | |
| Yes | 9.62 (2.12-17.12) | 0.41 | 5.77 (1.28-10.25) | 0.30 |
| No | 4.35 (2.66-6.88) |  | 2.05 (1.31-3.23) |  |
| **Complications** |  | | | |
| Yes | 4.53 (2.69-6.65) | 0.11 | 2.76 (1.34-5.36) | 0.10 |
| No | 4.33 (2.65-6.99) |  | 2.03 (1.30-3.09) |  |
| **Valve Type** |  | | | |
| Edwards | 4.35 (2.35-7.41) | 0.94 | 2.05 (1.24-3.25) | 0.49 |
| Medtronic | 4.34 (2.94-6.18) |  | 2.03 (1.45-3.23) |  |
| **Horizontal Aorta** |  |  |  |  |
| Yes | 4.78 (4.06-7.31) | 0.17 | 2.06 (1.18-2.71) | 0.45 |
| No | 4.33 (2.61-6.88) |  | 2.05 (1.30-3.25) |  |

**Table 4:** **Multivariable regression analysis for operator 1 and 2 radiation exposure including fluoroscopy time**

**Operator 1**

| **Variable** | **Parameter Estimate*** | **95% Confidence Intervals⁺** | **p-value** |
| --- | --- | --- | --- |
| **Obesity** | **2.74** | **2.37-3.22** | **< 0.0001** |
| **Fluoroscopy Time** | **2.03** | **2.02-2.04** | **< 0.0001** |

Multivariable regression analysis using log-transformed values of radiation for operator 1. * Parameter estimates derived from the coefficients from the log-transformed data by exponentiating the coefficients.

⁺ 95% confidence intervals were also obtained from the above-mentioned formula after adding and subtracting the value of 1.96 times the standard error to the log-transformed data.

**Operator 2**

| **Variable** | **Parameter Estimate*** | **95% Confidence Intervals⁺** | **p-value** |
| --- | --- | --- | --- |
| **Obesity** | **2.66** | **2.29 - 3.12** | **0.0001** |
| **Fluoroscopy time** | **2.03** | **2.02 - 2.04** | **< 0.0001** |
| **Complications** | **2.75** | **2.20 - 3.56** | **0.0045** |

Multivariable regression analysis using log-transformed values of radiation for operator 1. * Parameter estimates derived from the coefficients from the log-transformed data by exponentiating the coefficients.

⁺ 95% confidence intervals were also obtained from the above-mentioned formula after adding and subtracting the value of 1.96 times the standard error to the log-transformed data.
